# Exploring engagement with a web-based self-directed psychoeducational program for advanced cancer patients and their caregivers (iFOCUS): A sub study analysis of the DIAdIC trial

**DOI:** 10.1177/02692163261424464

**Published:** 2026-04-21

**Authors:** Ulrik Sørensen Schmidt, Romy Van Rickstal, Vincent Van Goethem, Evi Bakker, Monika Pilch, Michael Connolly, Paul D’Alton, Catherine Jordan, Philip J. Larkin, Peter May, Katherine Bristowe, Silvia de Leo, Aline De Vleminck, Joachim Cohen, Laurel Northouse, Peter Hudson, DIAdIC Author Group, Suzanne Guerin

**Affiliations:** 1 UCD School of Psychology, University College Dublin, Ireland; 2 Department of Public Health, University of Copenhagen, Denmark; 3 End-of-Life Care Research Group, Vrije Universitiet Brussel & Ghent University, Belgium; 4 Department of Public Health, Erasmus MC, University Medical Center Rotterdam, The Netherlands; 5 UCD School of Nursing, Midwifery & Health Systems & Our Lady’s Hospice and Care Services, Dublin, Ireland; 6 Palliative and Supportive Care Service, Lausanne University Hospital & University of Lausanne, Switzerland; 7 Centre for Health Policy & Management, Trinity College Dublin, Dublin, Ireland; 8 Cicely Saunders Institute of Palliative Care, Policy Rehabilitation, King’s College London, UK; 9 Cicely Saunders Institute of Palliative Care, Policy & Rehabilitation, Florence Nightingale Faculty of Nursing, Midwifery & Palliative Care, King’s College London, UK; 10 Azienda Unità Sanitaria Locale, IRCCS di Reggio Emilia, Italy; 11 End-of-life care research group, Vrije Universiteit Brussel (VUB) & Ghent University, Belgium; 12 School of Nursing, University of Michigan, USA; 13 Centre for Palliative Care, St Vincent’s & The University of Melbourne, Australia; 14 End of Life Research Group, Vrije University, Brussels, Belgium

**Keywords:** palliative care, cancer, caregivers, telemedicine, internet-based intervention, intervention adherence, randomized controlled trial, process evaluation

## Abstract

**Background:**

Supporting psychoeducational functioning for advanced cancer patients and informal caregivers (dyads) can be challenging. Self-managed digital interventions offer a possible cost-effective alternative but often face low engagement.

**Aim:**

To examine engagement with the iFOCUS intervention and to understand why it failed to improve the expected outcomes.

**Design:**

iFOCUS, a four-session digital self-managed 12-week intervention for dyads, was tested in six European countries as part of a randomized controlled trial. However, it showed no effects on outcomes. Using process evaluation data with 121 dyads, measures of engagement were examined to identify how engagement predicts dropout and to identify associations with patient/caregiver characteristics, post-intervention outcomes, and patient/caregiver evaluation of the intervention.

**Results:**

Measures of engagement showed variation in associations with participant characteristics. Lower engagement with the intervention among dyads at the outset, was associated with greater odds of dropout and engagement with the intervention decreased as the dyads progressed. Patients’ level of education and caregivers’ baseline emotional function, self-efficacy, dyadic coping, and knowledge of cancer were associated with engagement. However, there was no consistent evidence that engagement was associated with outcomes or evaluation of the intervention.

**Discussion:**

While we identified several associations between engagement and patient/caregiver characteristics, there were inconsistencies in the extent and nature of the associations, with more evidence that baseline characteristics impacted engagement, than evidence that engagement impacted on outcomes. However, using multiple measures of engagement did provide additional insights. A key recommendation from this study is the need for consideration of assessments of engagement for digital interventions.

**What is already known about the topic?**
Digital interventions offer a possible cost-effective intervention to promote outcomes for individuals affected by cancer, including patients and caregivers, but they often face low engagement.
Some common measures of engagement, such as time engaged with the intervention, show limitations and may not fully explain engagement with the intervention.
**What this paper adds**
This study demonstrates the benefits of more nuanced measures of engagement with digital interventions. The study goes beyond single variable measurement and provides additional metrics for assessing engagement, which uses routine data gathered by digital platforms.
This nuanced measurement of engagement with the digital iFOCUS intervention, highlights relationships with baseline characteristics, as well as some predictive influence on retention and post-intervention outcomes.
**Implications for practice, theory, or policy**
The findings support the need to reconsider how engagement is conceptualized and measured in studies evaluating digital interventions.

## Introduction

Patients with cancer often experience physical and psychosocial symptoms. Informal caregivers also face a significant caregiving burden, which can affect their own health.^1^ While programmes addressing patients and caregivers as a dyads exist,^2^ they are often costly and as a result, seldomly implemented at large scale.^3^ At the same time, digital interventions have been shown to positively impact several outcomes including coping, self-efficacy, quality of life and more.^3–6^

Digital dyadic interventions offer an alternative, as the absence of human delivery makes them cheaper and more scalable.^7^ Nevertheless, a review by Shaffer et al.^8^ of dyadic psychosocial eHealth interventions identified that 77% of the digital interventions depended to some degree on human intervention. This suggests a need for self-managed digital interventions that effectively support both parties simultaneously.

If effects are expected from digital interventions with psychosocial/psychoeducational purposes, a crucial question is to what extent people engage with them. While the definition of engagement varies, Nahum-Shani et al.^9^ report that in health research, engagement is a measure of the actions patients/caregivers take to improve their health. Nonetheless, research on such engagement is inconsistent.^9^ While various forms of digital interventions for various target groups have been evaluated,^5,6,10,11^ most research focuses on outcomes rather than engagement with these interventions. When engagement is considered, evidence suggests that aspects of the technical design of ehealth resources and the perception of therapeutic content are important,^12^ and the level of engagement can represent an important metric of dose with implications for effectiveness.^13^ Examining engagement is therefore crucial to increase our understanding of how these interventions work, and why they sometimes fail.

A systematic review of digital behaviour change interventions summarizes that psychological characteristics such as self-efficacy, and demographic characteristics such as age, gender, education, and computer literacy are associated with intervention engagement.^14^ Several studies have examined participants’ engagement with digital interventions by analysing data on retention, completion of sessions, and time spent on materials (e.g. Lambert et al.^15^). However, these measures have limitations.^16,17^ For instance, digital interventions often include informational and educational components, and measuring the time spent on these, does not necessarily help us understand how knowledge or self-efficacy are gained.^16,17^ A need for research that combines different measures of engagement has been suggested.^17^

The iFOCUS program is a dyadic psychoeducational digital intervention designed to be completed simultaneously by patients with advanced cancer and their informal caregiver. The development process drew on existing programmes, with input from professional and patient representatives to ensure the appropriateness of the intervention.^18^ The intervention was evaluated in an international three-arm randomized controlled trial (RTC), the DIAdIC trial.^19^ The iFOCUS intervention showed no significant effect on the primary endpoints (emotional functioning and self-efficacy).^20^

The aim of this study is to understand why the intervention failed to improve the expected outcomes, by:

(1) Examining dyads’ levels of engagement throughout the intervention.
(2) Exploring the relationship between initial engagement with the intervention and retention.
(3) Examining how engagement is associated with patient and caregiver characteristics, outcomes, and evaluation of the intervention.

## Methods

### Research design

We conducted a process and implementation evaluation within the DIAdIC trial. The methodology, including inclusion and exclusion criteria are described elsewhere.^19^ In this study, we focus on the implementation evaluation of the digital intervention arm, that is, the iFOCUS intervention.

### Participants

The population examined in this paper is 121 patient/caregiver dyads, recruited from six European countries and randomized to receive the iFOCUS intervention. Patients had advanced cancer, were no longer receiving curative treatment, but did not have a life expectancy of less than 3 months. The caregiver was the primary informal caregiver, as identified by the patient. Both parties had to have access to and be familiar with the internet and a computer/tablet.

### The iFOCUS intervention

iFOCUS is a self-managed psychoeducational intervention completed by dyads on a computer/tablet. Spread over four sessions completed within 12 weeks, the intervention addresses family involvement, outlook, coping strategies, uncertainty reduction, and symptom management through various psychoeducational strategies (see Van Goethem et al.^18^ and De Vleminck et al.^20^ for additional information). The content of the intervention includes videos (with transcriptions available), written information, strengths-and-needs assessments, tailored feedback based on these, and questions and exercises aimed at both parties. Dyads were expected to complete the entire intervention together.^18^ There was no interaction with dyads as part of the intervention. Members of the research team were available to support dyads with technical difficulties in accessing the online material. Engagement and completion were monitored automatically, with prompts issued by email when dyads were due to engage with the next section of the website.

### Data collection

Data were collected through two methods:

(1) Tracking and recording of engagement.

As dyads completed the iFOCUS intervention, routine data were stored, which tracked: initiation of the session (yes/no); seconds spent on each page; clicks on clickable content; responses to questions presented; and whether they started videos. If dyads dropped out in the middle of the session, their engagement data would be logged as missing from the point that they dropped out. Due to challenges with time tracking on random pages, some data had negative values, which were recoded as missing data.

(2) Questionnaires completed by patients and caregivers allocated to the iFOCUS intervention at baseline and post-intervention (12 weeks after baseline). Additional details are reported in the study protocol.^19^

### Measures

Miller et al.’s^21^ framework informed the analytical process and selection of engagement measures and variables.

We identified four measures of engagement:

(1) Time spent on iFOCUS, in the form of average seconds per page.
(2) Clicks, in the form of percentage of clickable content clicked.
(3) Video engagement, in the form of a video watch and completion score (video score). The video score attributed was respectively 0, 1 or 2 (0 if not started, 1 if started but not entirely watched, 2 if entirely watched). The video score was first calculated for each individual video page, and a total video score was then calculated for the session.
(4) Dropout, as an indicator of continuation to receive or not receive the intervention, dichotomized into 0; completion of two or fewer sessions, and 1; completion of three or four sessions.

From the baseline data, primary outcome measures (emotional function and self-efficacy) and two secondary outcome measures – Ways of Giving Support and Dyadic Coping Inventory, which relate to the dyads’ communication – were used. Emotional functioning was measured using 10 items from the EORTC, while self-efficacy was assessed using the 17-item Lewis’ Cancer self-efficacy scale.^19,20^ These measures were calculated as numerical scales. Additionally, sociodemographic variables (age, gender, education, relationship of the dyad), a measure of IT competency, and dyads’ knowledge of the cancer^20^ were included. These variables were categorized as binary, with ‘low-medium’ and ‘high’ groups.

Post-intervention questionnaire data were also used, specifically the two primary outcome measures and the two secondary outcome measures. Additionally, patient and caregivers’ responses to four evaluation questions as factors potentially impacted by engagement; ‘How useful was the programme to meeting your needs?’, ‘To what extent were you satisfied with the programme?’, ‘The information I received in the programme was important to me.’, and ‘The programme took too much time’. The evaluation questions were categorized as ternary variables with negative, neutral and positive response categories.

### Statistical analyses

Data analysis was completed in R-studio 2024.04.2.

Descriptive analysis generated mean and standard deviation (sd) for average seconds per page, percentage of clickable content clicked, and video score. Univariate logistic regression examined whether engagement, measured at Session 1, predicted dropout. Kruskal-Wallis tests were used to compare if engagement was significantly different across the four sessions, and Wilcoxon tests were used for session comparison. Correlation tests examined association between engagement and outcomes at baseline and follow-up. Kruskal-Wallis rank sum tests examined associations between engagement and baseline characteristics and evaluation of the intervention. The baseline and follow-up tests were completed using the engagement data from Session 1 and 4, separately. Unless otherwise stated, the effect directions for Kruskal-Wallis tests were assessed based on differences in medians.

Throughout this paper, a *p*-value of 0.025 or below was set as the level of statistical significance, taking into consideration the number of tests being conducted.

### Ethics

Dyads gave written consent at enrolment. On the iFOCUS platform, dyads were informed about routine data recording, their right to withdraw, and contact details for local data managers. Ethics approval was granted in participating countries (e.g. Belgium BC-08591, B6702020000776, Ghent University Hospital on October 26, 2020).

## Results

Characteristics of the trial participants have been described elsewhere,^20^ however summary data for the iFOCUS participants are presented in Table 1 and relevant descriptive analyses are include in Table 1a (Supplemental Material).

**Table 1.** Patients and caregivers demographic characteristics, n = 121.

Demographic characteristics	Patient		Caregiver	
Age	*n*	%	*n*	%
<66 years old	77	63.6	88	72.7
⩾66 years old	43	35.5	32	26.4
Missing (removed)	1	0.8	1	0.8
Gender				
Male	58	47.9%	39	32.2
Female	63	52.1	82	67.8
Other/prefer not to answer	0	0	0	0
IT-competences				
Low-medium	30	24.79	27	22.31
High	91	75.21	94	77.69
Level of education				
Low-middle	67	55.38	66	54.55
High	53	43.80	53	43.80
Prefer not to answer/missing (removed)	1	0.83	2	1.65
Knowledge of cancer				
Low-medium	51	42.15	74	61.16
High	69	57.02	47	38.84
Missing (removed)	1	0.83	0	0
	Mean	SD	Mean	SD
Emotional function	48	8.74	46.3	8.6
Self-efficacy	121.4	34.81	113.3	28.84
Dyadic coping inventory	3.07	0.68	2.92	0.66
Ways of giving support	4.21	0.69	4.18	0.57
Relation of caregiver to patient	*n*		%	
Spouse/partner	87		71.9	
Parent/sibling/child/other relative/friend/other	33		27.3	
Missing (removed)	1		0.8	

### Measures of engagement

As seen in Table 2, Session 1 had the most content of the four sessions, the highest total minutes spent, the highest average seconds per page, the greatest percentage of clickable content clicked, the greatest percentage of videos started, and the highest video score across all sessions. The four sessions were similar in terms of clickable elements, number of videos, number of questions, and percentage of questions answered.

**Table 2.** Content and engagement (time, percentage of content clicked and video score) by session.

Variable/measure	Session 1	Session 2	Session 3	Session 4
Dyads started session (*n*)	121	105	87	78
Number of set pages (*n*)	39	30	29	32
Number of clickable elements (*n*)	15	15	15	13
Number of videos (*n*)	8	8	9	6
Number of questions (*n*)	7	7	6	7
Total minutes spent on session, mean (sd)	48 (22)	37 (17)	29 (15)	29 (18)
Average seconds per page, mean (sd)	73.9 (34.5)	74.1 (34.4)	60.2 (30.7)	55.2 (32.9)
% of clickable content clicked,^a^ mean (sd)	93.4% (15)	89.1% (20)	80.17% (19.21)	88% (13.8)
% of videos started, mean (sd)	89.67% (21.8)	81.9% (30.9)	76.5% (32.6)	74.2% (33.5)
% of questions answered, mean (sd)	97.8% (12.9)	97% (13)	99% (8.9)	99.8% (1.6)
% of extra content downloaded, mean (sd)	39.7% (42.8)	27% (29.3)	33.1% (35.7)	68% (47)
Video score, mean (sd)	1.58 (0.44)	1.44 (0.61)	1.36 (0.60)	1.23 (0.60)

a Excluding extra content downloaded.

To understand how initial engagement influences retention, we examined the relationship between engagement at Session 1 and dropout. Thirty-five (28.83%) dyads completed just two or fewer sessions, while the remaining dyads (71.07%, *n* = 83) completed three or more sessions. Logistic regression examining the association between engagement and dropout, found that the percentage of clickable content clicked in Session 1 was a significant predictor of retention (Odds ratio = 0.965, *p* = 0.009). The more dyads engaged during Session 1, the less likely they were to drop out of the intervention. Average seconds per page (Odds ratio = 1.0002; *p* = 0.76) and video score (Odds ratio = 0.38; *p* = 0.03) in Session 1 were not associated with dropout.

As evident from the results of the Kruskal-Wallis test in Table 3, average seconds per page differed significantly across the four sessions. Using a pairwise Wilcox test, significant differences were found for both Session 1 and 2 when compared with Session 3 and 4. The negative difference in median, indicate that average time dyads spent per page, decreased the further along in the intervention they got. A similar pattern is seen for the percentage of clickable content clicked, as well as the video score. For the remaining analyses, only Session 1 and 4 engagement data are used, since they consistently differ in engagement and represent the start and end of the intervention.

**Table 3.** Comparison of engagement across sessions. Kruskal-Wallis (KW) tests for overall difference and pairwise Wilcox tests (WT) for session level comparison. Difference between sessions is indicated with difference in mean.

Engagement by session	Session 1		Session 2		Session 3	
	Difference in median	Adjusted *p*-value	Difference in median	Adjusted *p*-value	Difference in median	Adjusted *p*-value
Average seconds spent per page	KW, *p*-value: <0.001					
Session 2	Mean diff: 8.9	WT, *p*-value: 0.70				
Session 3	Mean diff: −6.2	WT, *p*-value: 0.011	Mean diff: −15.1	WT, *p*-value: 0.002		
Session 4	Mean diff: −17.5	WT, *p*-value: <0.001	Mean diff: −26.4	WT, *p*-value: <0.001	Mean diff: −11.3	WT, *p*-value: 0.069
% Of clickable content clicked	KW, p-value: <0.001					
Session 2	Mean diff: 0.00	WT, *p*-value: 0.305				
Session 3	Mean diff: −12.5	WT, *p*-value: <0.001	Mean diff: −12.5	WT, *p*-value: <0.001		
Session 4	Mean diff: −7.7	WT, *p*-value: <0.001	Mean diff: −7.7	WT, *p*-value: 0.001	Mean diff: 4.8	WT, *p*-value: <0.001
Video score	KW, p-value: < 0.001					
Session 2	Mean diff: 0.00	WT, *p*-value: 0.807				
Session 3	Mean diff: −0.14	WT, *p*-value: <0.001	Mean diff: −0.14	WT, *p*-value: 0.005		
Session 4	Mean diff: −0.25	WT, *p*-value: <0.001	Mean diff: −0.25	WT, *p*-value: <0.001	Mean diff: −0.11	WT, *p*-value: 0.046

### Patterns in engagement by baseline characteristics of patient and caregivers

Table 4 presents the tests examining associations of engagement with baseline outcomes and characteristics.

**Table 4.** Correlation and Kruskal-Wallis tests examining patient and caregiver baseline characteristics associated with engagement during Session 1 and 4. Correlation tests was completed for emotional function, self-efficacy, Dyadic Coping Inventory and Ways of Giving Support. Kruskal-Wallis tests was completed for age, gender, level of education, Knowledge of cancer, IT-competences and relation of caregiver to patient. Test for the relation of caregiver to patient is presented under the caregiver row but covers both the patient and caregiver.

Baseline characteristics	Session 1						Session 4					
	Patient			Caregiver			Patient			Caregiver		
	Seconds per page, *p*-value	Click %, *p*-value	Video score, *p*-value	Seconds per page, *p*-value	Click %, *p*-value	Video score, *p*-value	Seconds per page, *p*-value	Click %, *p*-value	Video score, *p*-value	Seconds per page, *p*-value	Click %, *p*-value	Video score, *p*-value
Emotional function	0.30	0.14	0.71	0.32	0.85	0.95	0.14	0.46	0.77	0.44	**0.01** ^b^	0.04
Self-efficacy	0.50	0.66	0.11	0.66	0.23	0.45	0.17	0.69	0.63	0.37	**0.01** ^b^	0.05
Dyadic coping inventory	0.19	0.79	0.24	0.48	0.08	**0.02** ^b^	0.46	0.91	0.50	0.67	0.59	0.72
Ways of giving support	0.81	0.07	0.17	0.40	0.11	0.06	0.97	0.29	0.26	0.64	0.46	0.98
Age	0.83	0.60	0.47	0.49	0.25	0.14	0.08	0.14	0.18	0.47	0.23	0.41
Gender	0.14	0.68	0.77	0.71	0.64	0.82	0.88	0.87	0.92	0.81	0.80	0.70
Level of education	0.39	0.97	0.82	0.64	0.92	0.70	**0.02** ^a^	0.12	0.03	0.48	0.53	0.57
Knowledge of cancer	0.66	0.82	0.97	**0.02** ^b^	0.23	0.04	0.97	0.26	0.15	0.69	0.46	0.57
IT-competences	0.30	0.74	0.22	0.50	0.61	0.68	0.67	0.83	0.55	0.72	0.67	0.20
Relation of caregiver to patient				0.03	0.91	0.74				0.21	0.57	0.08

For variables with significant p-value (marked in bold in the table) effect direction and difference in median is indicated with either.

a Positive effect size for correlation test/difference in median favouring the ‘high’ group.

b Negative effect size for correlation test/difference in median favouring the ‘low-medium’ group.

At Session 1, none of the patient variables were associated with engagement., Among caregivers, the Dyadic Coping Inventory score were significantly associated with the video score, with greater ability to cope associated with lower engagement. Additionally, greater caregiver knowledge of cancer was significantly associated with fewer average seconds per page.

At Session 4, higher level of education among patients was significantly associated with greater engagement, as average seconds per page. Caregivers’ emotional function at baseline was significantly associated with percentage of clickable content clicked, with greater emotional functioning associated with lower engagement. Finally, greater self-efficacy among caregivers was associated with lower engagement, as percentage of clickable content clicked.

### Relation between engagement and post-intervention outcomes and evaluation

Table 5 presents the associations of engagement with post-intervention outcomes and dyads’ evaluation of the intervention. Self-efficacy and video score for Session 4 was the only significant association, indicating those with higher self-efficacy had lower engagement.

**Table 5.** Correlation and Kruskal-Wallis tests examining association between patients and caregivers’ post-intervention outcomes and questions evaluating the intervention. Correlation tests was completed for emotional function, self-efficacy, Dyadic Coping Inventory and Ways of Giving Support. Kruskal-Wallis tests was completed for questions evaluating the intervention.

Post-intervention measures	Session 1						Session 4					
	Patient			Caregiver			Patient			Caregiver		
	Seconds per page, *p*-value	Click %, *p*-value	Video score, *p*-value	Seconds per page, *p*-value	Click %, *p*-value	Video score, *p*-value	Seconds per page, *p*-value	Click %, *p*-value	Video score, *p*-value	Seconds per page, *p*-value	Click %, *p*-value	Video score, *p*-value
Post-intervention outcomes												
Emotional function	0.37	0.72	0.67	0.87	0.54	0.33	0.10	0.50	0.26	0.19	0.04	0.11
Self-efficacy	0.71	0.62	0.34	0.37	0.22	0.14	0.44	0.66	0.86	0.27	0.09	**0.02** ^a^
Dyadic coping inventory	0.82	0.50	0.77	0.52	0.17	0.77	0.84	0.63	0.95	0.31	0.75	0.87
Ways of giving support	0.64	0.89	0.62	0.69	0.24	0.15	0.98	0.46	0.64	0.73	0.76	0.63
Questions evaluating the intervention												
Usefulness of iFOCUS	0.54	0.35	0.88	0.27	0.77	0.50	0.40	0.59	0.20	0.15	0.51	0.19
Satisfaction with iFOCUS	0.36	0.78	0.94	0.05	0.97	0.62	0.61	0.27	0.09	0.68	0.77	0.59
Importance of Information	0.47	0.69	0.99	0.21	0.79	0.30	0.77	0.24	0.35	0.59	0.71	0.35
The programme took too much time.	0.90	0.84	0.70	0.67	0.68	0.94	0.17	0.33	0.57	0.36	0.36	0.19

For variables with significant p-value (marked in bold in the table) effect direction and difference in median is indicated with either.

a Negative effect size for correlation test/difference in median favouring the ‘low-medium’ group.

## Discussion

### Key findings

We found that dyads’ engagement during Session 1 predicted dropout, and that dyads engaged significantly less in later sessions. In addition, there is variation across measures of engagement, and several associations between patient/caregiver characteristics and measures of engagement, highlighting the diversity of engagement with iFOCUS.

The findings from the wider trial show that iFOCUS failed to improve the expected outcomes.^20^ Our findings add that the dyads engaged less than expected, resulting in them not receiving the intervention as intended. As stated earlier, engagement is a measure of the actions patients/caregiver take to improve their health.^9^ If that action is less than what was anticipated when the intervention was developed, it will reflect low fidelity, and we can therefore not assume the intervention was received as planned. In addition, findings show that dyads engaged less over the course of the intervention, which further reduces the expected impact. This finding is consistent across our measures of engagement, and in line with previous research.^22^ Research has indicated a link between engagement and outcomes,^23,24^ possibly in a dose-response relationship.^25^ However, this study does not suggest a consistent and significant association between engagement and outcomes. A dose-response exploration of the association between engagement and outcomes might further help us understand this.

Additionally, we found that lower engagement in Session 1 predicts higher odds of dropout. Dyads, who from the beginning of the intervention take little action, are therefore more likely to receive even less of the intervention, ultimately receiving the intervention differently than planned. This aligns with findings from other studies on digital intervention.^22,26^

In terms of associations between engagement and baseline characteristics, there are some patterns of difference. Better emotional function, self-efficacy and Dyadic Coping Inventory score for caregivers were associated with lower engagement, while lesser knowledge of cancer among the caregiver, as well as higher education among the patient, was associated with higher engagement. This could indicate that dyads with well-functioning caregivers engaged less with iFOCUS, possibly because they did not see a need for additional support. However, our findings are inconsistent, with differences in association for patients and caregivers and in Session 1 and 4. Similarly, the associations between engagement, the patient’s education level, and the caregiver’s cancer knowledge suggest unclear findings, as both may reflect how well-informed the dyad is, but they influence engagement in different directions. As engagement differed significantly across sessions, it likely explains the differences in associations for Session 1 and 4. The differences between patients and caregivers might be explained by the fact that while we consider dyads one unit, it is made up of two people – which are likely to be different, as one is a patient and one is a caregiver.

Finally, the findings indicate no association between engagement and how dyads evaluated iFOCUS, indicating engagement does not predict experience with the intervention. Forthcoming papers exploring dyads’ experience with iFOCUS are expected to provide a clearer understanding of variations in engagement and impact.

These key findings suggest that iFOCUS did not encourage engagement, raising the question of whether the lack of significant effects reflects a failure of implementation or of the intervention itself. We argue it was an intervention failure, as it failed to do what it was designed to do – engage dyads. However, the design likely also undermined implementation – evident from declining engagement and retention as the intervention progressed.

### Strengths and limitations

A strength of this study is the use of several measures of engagement, as recommended by O’Brien and Lebow^17^ and Donkin et al.^27^ However, there are limitations. The video score fails to account for the fact that dyads might have chosen not to start the video and instead read the online transcript, which could not be tracked in this study. In addition, the time-based measures are limited in their ability to capture how knowledge or self-efficacy are gained.^16,17^ However, our findings are not solely based on time as a measure of engagement. In retrospect, the study would have benefitted from audio recordings of the interaction between the dyad while they were in session, which would have allowed for a more nuanced understanding of these interactions and their engagement with the intervention.

Additionally, the option to use post-intervention questionnaire-based measures of engagement could have contributed with a more nuanced assessment of engagement.^28–30^ Some elements were nevertheless covered in the evaluation questions, for example, the question ‘The programme took too much time’ covered aspects of engagement related to time.

A final limitation is the lack of adjustment for confounders in the statistical analysis, as most of the tests are limited to examining the relationship between two variables. Alternative tests were not possible due to the data not meeting the required assumptions.

### Implications

Research suggests that dyadic eHealth interventions that incorporate human intervention improve health outcomes,^8^ and that such combination can positively affect engagement.^24^ However, the added human element and associated resource demands can limit widespread implementation. Self-managed digital interventions that promote engagement and result in similar improvements are therefore relevant.

One approach that has proven effective among digital interventions is tailoring to provide personalized content and increase relevance for participants.^8,23,24^ iFOCUS has some tailoring, however the lack of significant effect from the intervention^19^ and our findings, suggest that the intervention could have benefitted from additional tailoring. For example, if dyads skipped videos or had a preference towards non-video-based content, they could, with improved tailoring, have been presented with alternative content providing similar messages. To assist with further tailoring, artificial intelligence enhanced content (e.g. chatbot-models), which have shown promising results,^31–33^ might prove fruitful.

Finally, we recommend that measures of engagement become integrated elements of digital interventions, as they are with iFOCUS, as this allows for an understanding of why an intervention fails or succeeds. Throughout this paper, we have presented suggestions on how to measure and analyse engagement.

## Conclusion

In conclusion, we examined engagement with the iFOCUS intervention using different measures of engagement. We found that the less dyads engaged with the intervention in the beginning, the greater the odds of dropout. Additionally, we found that dyads engaged less as they progressed through the intervention. We identified associations indicating that the caregiver’s baseline emotional function, self-efficacy, the Dyadic Coping Inventory score, knowledge of cancer, and the patient’s level of education are associated with engagement. An important recommendation from this study is the need for more nuanced understanding of engagement and the need to measure and analyse engagement in digital dyadic interventions.

## Supplemental Material

sj-docx-1-pmj-10.1177_02692163261424464 – Supplemental material for Exploring engagement with a web-based self-directed psychoeducational program for advanced cancer patients and their caregivers (iFOCUS): A sub study analysis of the DIAdIC trial Supplemental material, sj-docx-1-pmj-10.1177_02692163261424464 for Exploring engagement with a web-based self-directed psychoeducational program for advanced cancer patients and their caregivers (iFOCUS): A sub study analysis of the DIAdIC trial by Ulrik Sørensen Schmidt, Romy Van Rickstal, Vincent Van Goethem, Evi Bakker, Monika Pilch, Michael Connolly, Paul D’Alton, Catherine Jordan, Philip J. Larkin, Peter May, Katherine Bristowe, Silvia de Leo, Aline De Vleminck, Joachim Cohen, Laurel Northouse, Peter Hudson and Suzanne Guerin in Palliative Medicine
