# Supplementary material for: Exploring engagement with a web-based self-directed psychoeducational program for advanced cancer patients and their caregivers (iFOCUS): A sub study analysis of the DIAdIC trial
Source: Palliat Med. 2026 Apr 21;40(5):644–54. doi: 10.1177/02692163261424464 (PMC13136541; doi:10.1177/02692163261424464)
Supplement: sj-docx-1-pmj-10.1177_02692163261424464 – Supplemental material for Exploring engagement with a web-based self-directed psychoeducational program for advanced cancer patients and their caregivers (iFOCUS): A sub study analysis of the DIAdIC trial [file sj-docx-1-pmj-10.1177_02692163261424464.docx]

**Supplementary Material**

*Table 1a: Post-intervention outcomes and patients’ and caregivers’ evaluation of the intervention. Dyads that did not start the first session were excluded.*

|  | **Patient** | | **Caregiver** | |
| --- | --- | --- | --- | --- |
|  | **Mean** | **sd** | **Mean** | **sd** |
| Emotional function | 49.66 | 8.53 | 47.32 | 7.69 |
| Self-efficacy | 120.60 | 32.90 | 116.00 | 31.34 |
| Dyadic Coping Inventory | 3.04 | 0.71 | 2.87 | 0.71 |
| Ways of Giving Support | 4.24 | 0.70 | 4.21 | 0.65 |
|  |  |  |  |  |
| *Questions evaluating the intervention* | **n** | **%** | **n** | **%** |
| **Usefulness of iFOCUS** |  |  |  |  |
| Not useful | 17 | 19.77% | 20 | 24.39% |
| Neutral | 51 | 59.30% | 47 | 57.32% |
| Useful | 18 | 20.93% | 15 | 18.29% |
|  |  |  |  |  |
| **Satisfaction with iFOCUS** |  |  |  |  |
| Dissatisfied | 10 | 11.76% | 15 | 18.07% |
| Neutral | 53 | 62.35% | 50 | 60.24% |
| Satisfied | 22 | 25.88% | 18 | 21.69% |
|  |  |  |  |  |
| **Importance of information** |  |  |  |  |
| Disagree | 21 | 24.42% | 24 | 28.92% |
| Neutral | 51 | 59.30% | 47 | 56.63% |
| Agree | 14 | 16.27% | 12 | 14.46% |
|  |  |  |  |  |
| **The programme took too much time.** |  |  |  |  |
| Disagree | 44 | 51.76% | 39 | 46.99% |
| Neutral | 30 | 35.29% | 32 | 38.55% |
| Agree | 11 | 12.94% | 12 | 14.46% |
